# Inactivation of KhpB (EloR/Jag) in *Lactococcus cremoris* increases uptake of the compatible solute glycine-betaine and enhances osmoresistance

**DOI:** 10.1128/aem.00914-25

**Published:** 2025-09-17

**Authors:** Yuwei Xiang, Huong Thi Pham, Yosephine Gumulya, Zhao-Xun Liang, Esteban Marcellin, Mark S. Turner

**Affiliations:** 1 School of Agriculture and Food Sustainability, University of Queensland 1974, Brisbane, Queensland, Australia; 2 Food and Beverage Accelerator (FaBA), The University of Queensland 1974, Brisbane, Queensland, Australia; 3 The University of Danang, University of Science and Technology, Da Nang, Vietnam; 4 School of Biological Sciences, Nanyang Technological University 54761, Singapore, Singapore; 5 Australian Institute for Bioengineering and Nanotechnology, University of Queensland 1974, Brisbane, Queensland, Australia; Washington University in St. Louis, St. Louis, Missouri, USA

**Keywords:** *Lactococcus cremoris*, osmotic stress resistance, cyclic-di-AMP, KhpB, glycine-betaine, BusAA-AB

## Abstract

The second messenger cyclic-di-AMP (c-di-AMP) is a signaling molecule widely present in gram-positive bacteria, where it regulates osmotic resistance by controlling potassium and compatible solute transport. Our previous studies using a *Lactococcus cremoris* model strain demonstrated that mutants with elevated c-di-AMP can overcome osmosensitivity through mutations enhancing potassium transporter activity. To identify additional mechanisms that enhance osmoresistance, we conducted a salt-resistance suppressor screen in an industrial *L. cremoris* strain. Using a spontaneous GdpP phosphodiesterase mutant with high c-di-AMP, we isolated salt-resistant suppressor mutants harboring six independent mutations in the *khpB* gene. These *khpB* mutants maintained elevated c-di-AMP levels comparable to the parental *gdpP* mutant. Inactivating *khpB* in wild-type and *gdpP* mutant laboratory *L. cremoris* strains similarly enhanced osmoresistance. KhpB (also known as EloR/Jag) is a putative RNA-binding protein, and its inactivation increased RNA transcript and protein expression of the glycine-betaine transporter BusAA-AB, elevating intracellular glycine-betaine uptake. Additionally, *khpB* disruption resulted in reduced cell size and enhanced secretion of native cell wall-degrading enzymes. Thus, KhpB likely acts as an indirect repressor of osmoresistance in *L. cremoris* by negatively regulating glycine-betaine transporter production.

**IMPORTANCE:**

*Lactococcus cremoris* is a model lactic acid bacterium and an industrially valuable fermentation starter culture. Similar to other gram-positive bacteria, *L. cremoris* utilizes the nucleotide messenger c-di-AMP to manage responses to osmotic stress. A suppressor screen aimed at restoring salt resistance in a high c-di-AMP mutant identified several independent mutations within the *khpB* gene. Our results demonstrate that *khpB* disruption elevates intracellular glycine-betaine concentrations, a prominent osmoprotectant. Notably, *khpB* inactivation also reduced cell size and enhanced the secretion of native cell wall-degrading enzymes. This study thus reveals KhpB as a negative regulator of osmotic stress resistance in *L. cremoris*, thereby expanding our understanding of bacterial osmoadaptation mechanisms.

## INTRODUCTION

*Lactococcus cremoris* is an acid-tolerant, non-sporulating, facultative anaerobic ovococcal-shaped gram-positive bacterium generally recognized as safe. *L. cremoris* is commonly used as a starter culture or naturally found in unfermented and fermented food products like raw milk, vegetables, fruits, cheese, yogurt, buttermilk, and sauerkraut (1–4). During its growth in diverse environments, *L. cremoris* encounters various stressors, including heat, oxidative stress, acid stress, starvation, and osmotic stress (5). Among these, osmotic stress resistance mechanisms are well characterized, with transporters responsible for importing the compatible solute glycine-betaine (BusAA-BusAB, also known as OpuA) and potassium (Kup) playing essential roles (6–10). The bacterial second messenger, cyclic-di-AMP (c-di-AMP), is a key regulator of osmotic stress resistance in *L. cremoris*. It binds directly to BusAA and the *busAA-busAB* transcriptional repressor BusR, leading to reduced intracellular glycine betaine uptake (6, 11). BusAA acts as the ATPase component of the transporter complex, while BusAB consists of the permease and substrate-binding components as a fusion protein. C-di-AMP also binds directly to KupA and KupB, inhibiting potassium uptake (12). The intracellular level of c-di-AMP in *L. cremoris* rapidly adjusts in response to external osmolarity changes, allowing the cell to modulate its turgor pressure accordingly (6).

In *L. cremoris*, c-di-AMP homeostasis is regulated by the diadenylate cyclase enzyme CdaA, which synthesizes c-di-AMP from two ATP molecules, and the phosphodiesterase enzyme GdpP, which breaks it down to pApA (13–16). Genetic mutations in *cdaA* or *gdpP* can lead to significant imbalances in c-di-AMP levels, adversely affecting bacterial growth. Mutants lacking functional *cdaA* are highly susceptible to cell wall-acting antibiotics and exhibit poor growth in low-osmolarity media, while mutants with defective *gdpP* display severe growth defects in high-osmolarity conditions (14–17). These phenotypes are likely due to dysfunctional osmolyte accumulation.

Previous studies using the well-characterized laboratory strain *L. cremoris* MG1363 identified several mutations that restored salt resistance in *gdpP* mutants (6, 15). Most mutations led to a reduction of c-di-AMP level, either through partial inactivation of the *cdaA* gene, restoration of *gdpP* activity, or increased nucleotide export via overexpression of a multidrug efflux pump (6, 15). The only mutations that both restored salt resistance and retained high intracellular c-di-AMP levels involved gain-of-function changes in the *kupB* gene, which increased potassium uptake (6). Notably, *L. cremoris* MG1363 naturally contains a truncated and non-functional *cdaR* gene, located immediately downstream of *cdaA* (13, 15). CdaR typically binds to and regulates CdaA activity (15, 18–21). In addition, MG1363 exhibits the phenotype-genotype disparity: while its genome classifies it as a stress-sensitive *cremoris* species, its phenotype resembles the stress-resistant *lactis* species, displaying growth at 4°C, 4% NaCl, and the ability to hydrolyze arginine, yet it is unable to grow in milk (22–26).

To expand our understanding of osmoresistance mechanisms in *L. cremoris*, we utilized an industrial *L. cremoris* strain (ASCC892185, referred to as WT-1 below) which contains an intact *cdaR* gene, exhibits a stress-sensitive species *cremoris* phenotype, and can grow in milk. Using a *gdpP* mutant of WT-1, we identified new mutations not found using the MG1363 strain background that led to the restoration of salt resistance (while maintaining a high c-di-AMP level). Six independent mutations were found in the *khpB* gene, which encodes a putative RNA-binding protein. In this study, we explored the role of *khpB* in osmoresistance at both transcriptional and protein levels, as well as its impact on cell morphology.

## RESULTS

### Suppressor mutants restore osmoresistance in an *L. cremoris gdpP* mutant without lowering the c-di-AMP level

To further expand our understanding of osmotic stress resistance mechanisms in *L. cremoris* and the role of c-di-AMP, we screened for suppressor mutants using a *gdpP* mutant (*gdpP*) of a non-model *L. cremoris* strain in different growth media (*n* = 7) with varying inhibitory concentrations of NaCl. A total of 160 colonies were isolated from streak plates, and 70 out of these 160 colonies were subsequently confirmed as being permanently NaCl resistant by a serial dilution spotting method. Previous work found that a large number of osmoresistant suppressors contained mutations in the *cdaA* gene, which led to a reduced c-di-AMP level in the *gdpP* mutant background (15). The *cdaA* gene was amplified by PCR from the osmoresistant suppressors and sequenced. Out of 70 osmoresistant suppressors, 27 contained mutations in the *cdaA* gene, including 23 substitution mutations, 3 frameshift mutations, and 1 nonsense mutation. To investigate c-di-AMP-independent mechanisms of osmoresistance, we selected 21 non-*cdaA* osmoresistant suppressors from the remaining 43 for analysis using whole-genome sequencing (WGS) (Table 1; Table S1). These 21 suppressors could be categorized into four different genetic groups based on their mutations (Table 1).

**TABLE 1:** Mutations identified using WGS in osmoresistant suppressors of *gdpP* that have an unaltered *cdaA* gene

Suppressor number	KhpB	Cell wall pellicle gene cluster (CWPS^*c*^)	FtsX	GreA	Other mutations
1, 2, 3^*a*^	K207 frameshift				
4	E173 frameshift				
5	G258 frameshift				
6, 7	D198 frameshift				Oligosaccharide flippase family protein RfbX^*b*^ (W128 stop codon), CBS domain YtoL^*b*^ (K243 frameshift)
8	R19 stop codon				
9, 10, 11, 12^*a*^	K122 frameshift				
13		WpsG (S245 frameshift)			
14		WpsF (E149 frameshift)			
15		WpsD (E27 stop codon)			
16		WpsF (S97 frameshift)			
17		WpsE (L223 frameshift)			
18		WpsA (A59V)			
19		WpsF (G174 frameshift)			
20		WpsF (K93 frameshift)			
21			A62D		
22			Deletion upstream of *ftsEX* (~117 bp)		
23				E18 stop codon	

^*a*^ Number code of individual suppressors with the same mutation(s).

^*b*^ Encoded protein names, from the NCBI website.

^*c*^ CWPS, cell wall polysaccharide.

**Fig 1:**
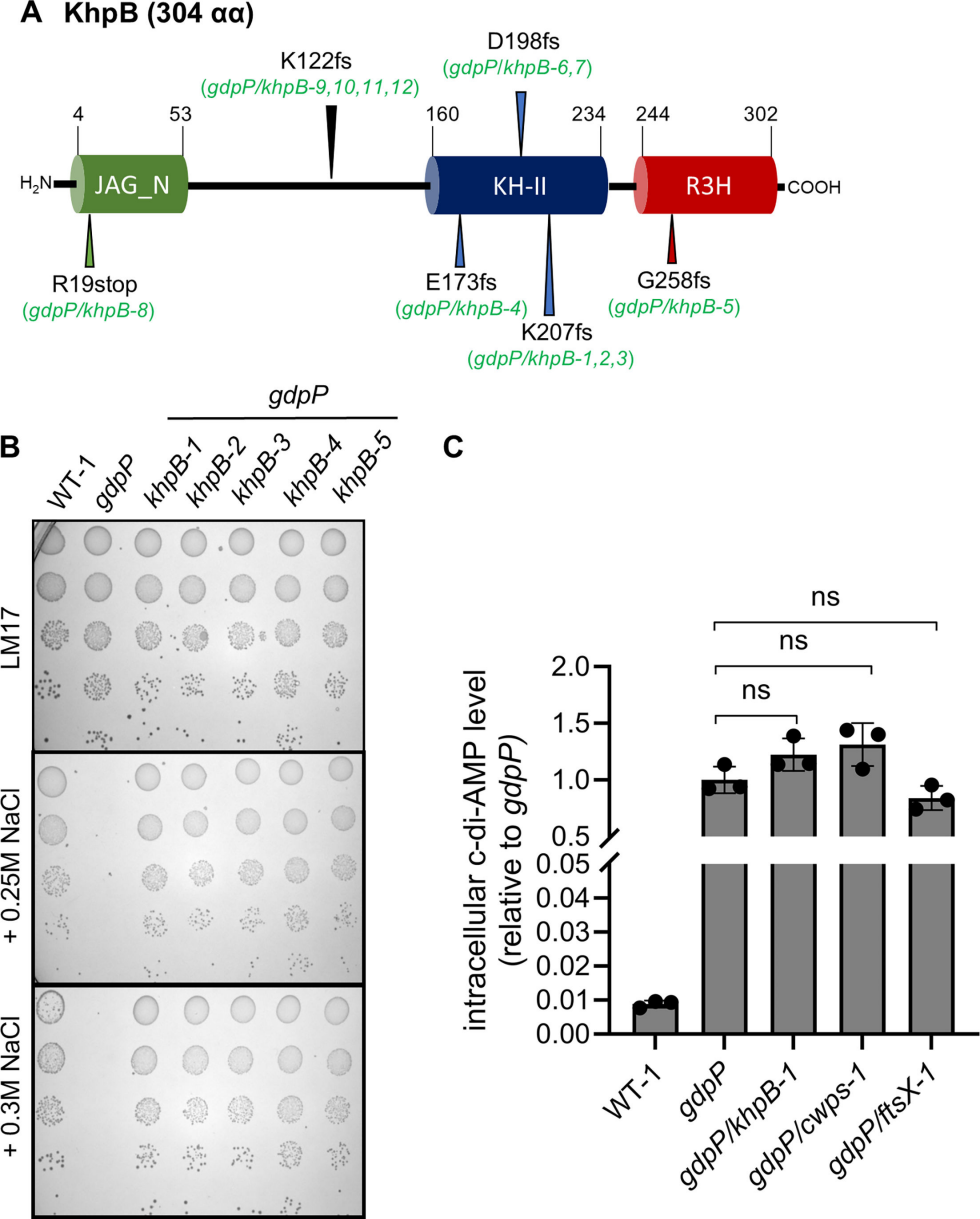
Salt-resistant suppressor mutant characterization. (**A**) Schematic representation of KhpB and the location of mutations in KhpB identified in the suppressor mutants of *gdpP*. The locations of the domains in the schematic figure were obtained from the Pfam database (KH is a putative RNA-binding domain with the signature motif GXXG; R3H contains the conserved arginine and histidine residues and is a putative ssDNA-binding domain). fs, frameshift mutation; stop, stop codon introduction. (**B**) Salt-resistance evaluation of *L. cremoris* WT-1, *gdpP*, and suppressors *gdpP*/*khpB-1* to *gdpP/khpB-5* obtained from *gdpP* using a serial dilution spotting assay. (**C**) Intracellular c-di-AMP levels in *L. cremoris* strains, including salt-resistant suppressors *gdpP/khpB-1*, *gdpP/cwps-1*, and *gdpP/ftsX-1*. Data are shown as mean ± standard deviation. One-way analysis of variance followed by a multiple comparison test was used for statistical analysis (*P* > 0.05) using three biological replicates. ns, not significant.

Several suppressors (*n* = 10) contained mutations in *khpB* (Fig. 1A; Table 1) involved in cell wall biosynthesis regulation. KhpB is an RNA-binding protein composed of an N-terminal Jag-N domain and RNA-binding KH and R3H domains (Fig. 1A) (27–29). This three-domain structure is highly conserved in Firmicutes, including *L. cremoris*, *Streptococcus pneumoniae*, *Bacillus subtilis*, *Lactiplantibacillus plantarum*, and *Clostridioides difficile*, but they have highly variable length spacer regions between the Jag and KH domains (27, 28). Sanger sequencing of *khpB* in additional suppressor mutants identified two more *khpB* mutations. In total, of the 12 *khpB* mutants identified, 11 had frameshift mutations and 1 had a stop codon (Fig. 1A). The second most common group of suppressors (*n* = 8) had mutations in the 22- or 23-gene cell wall pellicle gene cluster encoding machinery for the synthesis and export of a non-peptidoglycan cell wall polysaccharide (CWPS) (Table 1; Table S1). This rhamnose-containing CWPS is involved in cell wall biosynthesis and cell division and acts as a bacteriophage surface receptor (30–32). The third group of suppressors contained either a substitution mutation in *ftsX* (*ftsX-1*) or a 117 bp deletion upstream of *ftsEX* (*ftsX-2*) (Table 1; Table S1). FtsX is part of the FtsEX system involved in regulating peptidoglycan hydrolysis and cell division (33, 34). The final mutation occurred in GreA, which is a transcription elongation factor (Table 1; Table S1) (35).

C-di-AMP levels in representative suppressors from the three classes of mutants affecting cell wall functions (*gdpP*/*khpB-1*, *gdpP/cwps-1*, and *gdpP/ftsX-1*) were found to be as equivalently high as the parent strain (*gdpP*) (Fig. 1C). This suggests that salt resistance has been restored by means other than the alleviation of c-di-AMP-inhibited osmolyte transport systems.

### KhpB inactivation enhances osmoresistance in both the *L. cremoris gdpP* mutant and the wild-type background strain

**Fig 2:**
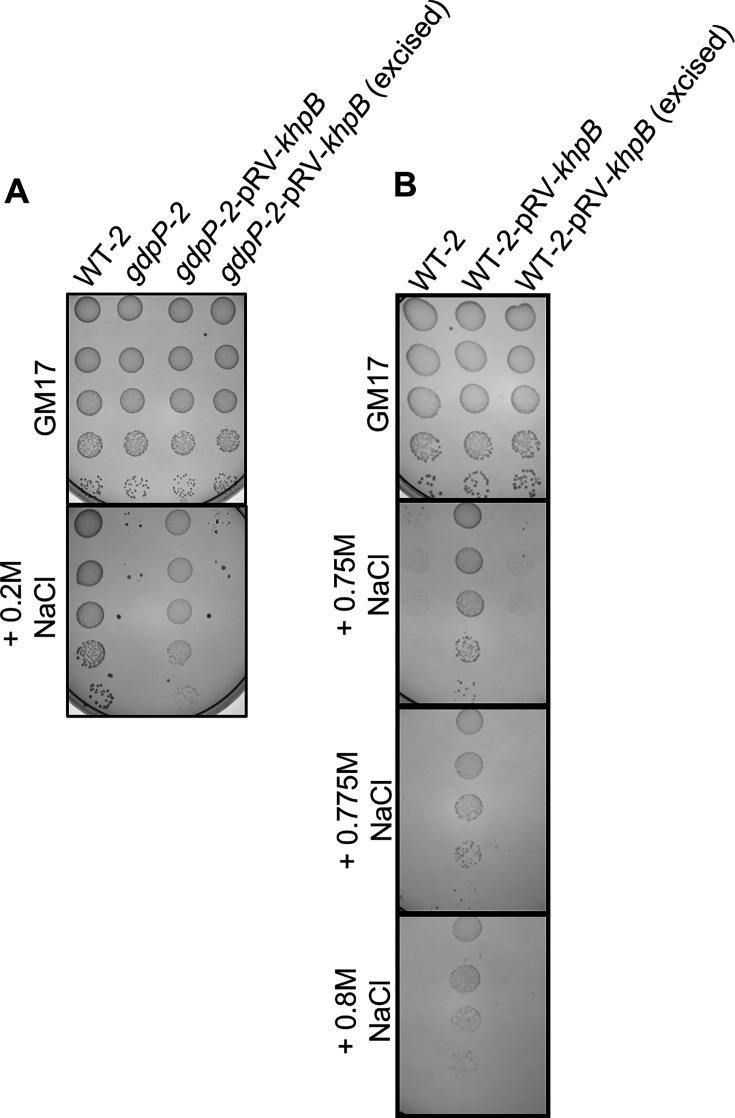
Salt resistance of isogenic *khpB* mutants of WT-2 and *gdpP-2*. (**A**) Salt-resistance evaluation of *L. cremoris* WT-2, *gdpP-2*, *gdpP-2* with *khpB* inactivated strain (*gdpP-2*-pRV-*khpB*), and a strain with the plasmid-excised from the *gdpP-2* with *khpB* inactivated strain (*gdpP-2*-pRV-*khpB* [excised]). (**B**) Salt-resistance evaluation of *L. cremoris* WT-2, WT-2 with *khpB* inactivated strain (WT-2-pRV-*khpB*), and a strain with the plasmid-excised from the WT-2 with *khpB* inactivated strain (WT-2-pRV-*khpB* (excised).

To further investigate the role of KhpB in osmoresistance in *L. cremoris*, we attempted to genetically manipulate the non-model industrial *L. cremoris* strain (WT-1) used in the suppressor screening. However, these efforts were unsuccessful. Instead, we were able to generate a *khpB* (*LLMG_RS00780 locus tag*) knockout mutant in the model laboratory strain, WT-2 (MG1363), and its high c-di-AMP *gdpP* mutant (*gdpP-2*). We showed that the inactivation of *khpB* using the suicide plasmid pRV300 in a *L. cremoris gdpP* mutant increases salt resistance (Fig. 2A). Excision of the plasmid reversed this effect, restoring salt sensitivity (Fig. 2A) and verifying that no other mutations were involved. Interestingly, *khpB* inactivation also increased salt resistance in the low c-di-AMP wild-type strain (WT-2) (Fig. 2B). Upon plasmid excision, salt resistance decreased to WT-2 level (Fig. 2B). These findings demonstrate that *khpB* inactivation improves osmoresistance across different *L. cremoris* strains, regardless of their c-di-AMP level.

### KhpB depletion enhances the transcriptional expression and protein abundance of the glycine betaine transporter (BusAA-AB), leading to increased intracellular glycine betaine levels

**Fig 3:**
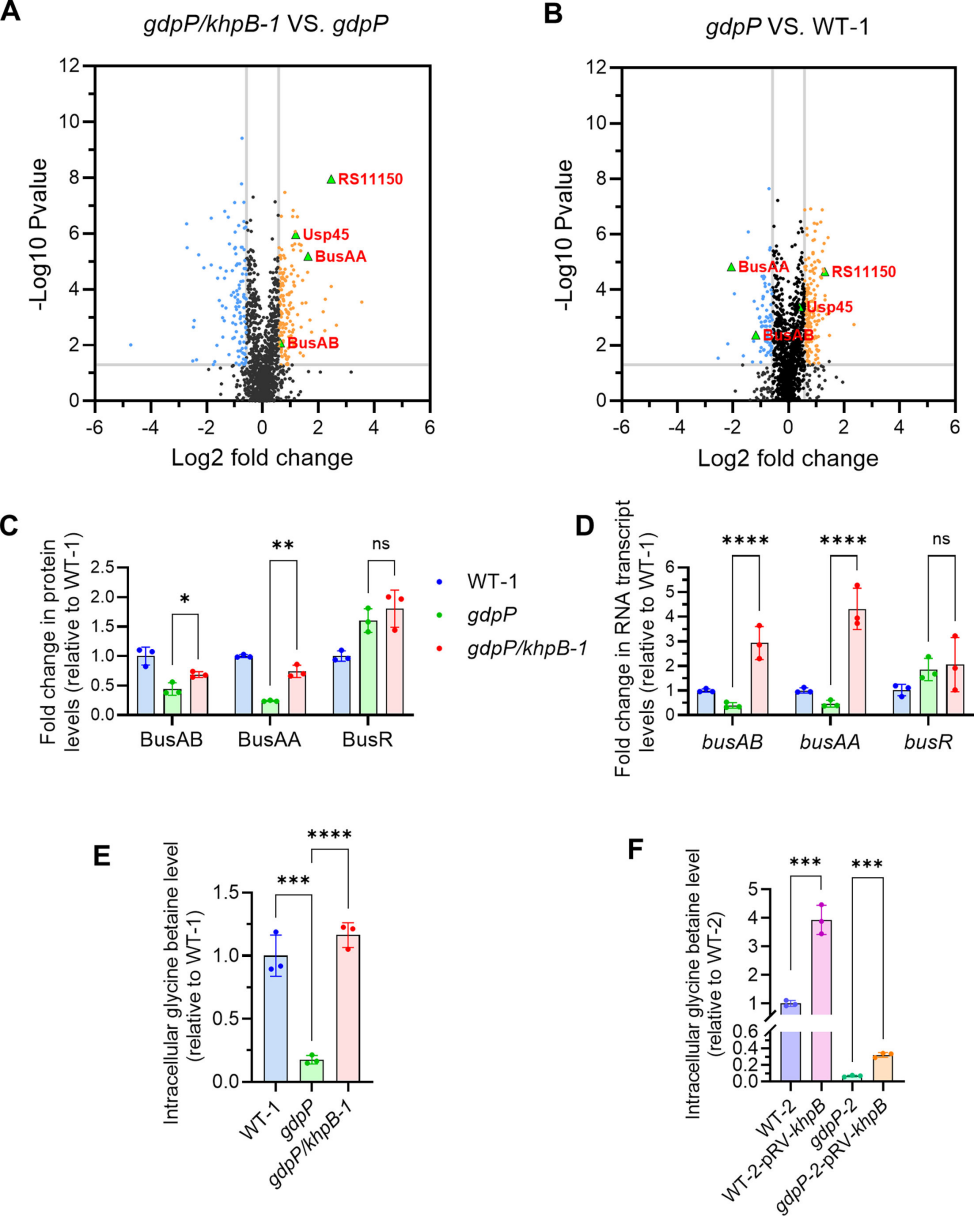
Upregulation of glycine betaine transporter (BusAA-AB) genes and protein abundance in the *khpB* mutant results in increased intracellular glycine betaine. Volcano plots show differential protein abundance in *gdpP/khpB-1* vs *gdpP* (**A**) and *gdpP* vs WT-1 (**B**). Volcano plots were generated using GraphPad Prism (version 10.2.2). The fold change cutoff was set at −0.58 ≤ log_2_(fold change) ≤ 0.58, with statistical significance defined as −log_10_(*P* value) ≥ 1.3 (*P* < 0.05). (**C**) Abundance of proteins BusAA, BusAB, and BusR in WT-1, *gdpP*, and *gdpP/khpB-1* from the proteomics data set. Data are shown as mean ± standard deviation (SD). Statistical analysis was performed using an unpaired two-tailed *t*-test (*P < 0.05, **P < 0.01) with three biological replicates. (**D**) Reverse transcription quantitative PCR analysis of RNA transcript levels of genes in WT-1 of *busAA*, *busAB*, and *busR* in WT-1, *gdpP*, and *gdpP/khpB-1*. Data are shown as mean ± SD. Two-way analysis of variance (ANOVA) followed by a multiple comparison test was used for the significant difference analysis (***P < 0.001, ****P < 0.0001) using three biological replicates. (**E**) Intracellular glycine betaine level in WT-1 (industrial strain ASCC892185), *gdpP*, and *gdpP/khpB-1*. Data are shown as mean ± SD. Ordinary one-way ANOVA followed by a multiple comparison test was used for the significant difference analysis (***P < 0.001, ****P < 0.0001) using three biological replicates. (**F**) Intracellular glycine betaine level in WT-2 (laboratory strain MG1363), WT-2-pRV*-khpB*, *gdpP-2*, and *gdpP-2*-pRV-*khpB*. Data are shown as mean ± SD. Unpaired two-tailed *t*-test was used for the significant difference analysis (***P < 0.001) using three biological replicates. ns, not significant.

KhpB from different bacteria has been found to bind and copurify with many RNA, including mRNA, tRNA, and small regulatory RNA (27, 36–38). Recent work in *Mycobacterium tuberculosis* reported that KhpB and its interaction partner, KhpA, are part of the RNA degradosome complex, which regulates the half-life of mRNA transcripts (29). We therefore hypothesized that mutations that inactivate *khpB* restore osmoresistance in *Lactococcus* by increasing the expression of genes involved in osmotic stress resistance, such as potassium and/or osmolyte transporters. To test this, we compared the proteome of the *gdpP*/*khpB-1* mutant with its parent strain *gdpP* (supplemental material). Either no significant change or reduced expression was observed in the *gdpP*/*khpB-1* mutant, relative to *gdpP*, for proteins involved in potassium transport (Kup [0.7-fold] and TrkH [1.0-fold]), glutamine transport (GlnP [0.5-fold] and GlnQ [1.0-fold]), oligopeptide transport (OppA [0.9-fold], OppB [0.4-fold], OppF [0.8-fold], and OppD [0.7-fold]), and putatively choline transport (OAS79_RS03005 [1.1-fold] and OAS79_RS03000 [1.0-fold]). However, the glycine-betaine transport system components were significantly more abundant in the *gdpP*/*khpB-1* mutant (BusAA [3.1-fold] and BusAB [1.6-fold]) (Fig. 3A). The level of the BusAA-AB transcriptional repressor BusR remained relatively constant at 1.1-fold. We also compared the proteome of the *gdpP* mutant relative to its parent strain (WT-1), and the BusAA-AB proteins were significantly less abundant (0.2-fold and 0.4-fold, respectively) in the *gdpP* mutant (Fig. 3B; supplemental material). From this proteomics analysis, it appears probable that repression of glycine-betaine transporter expression observed by high intracellular c-di-AMP is relieved upon *khpB* inactivation (Fig. 3C).

To determine if the changes in the glycine-betaine transporter expression level are also present at the mRNA level, we carried out reverse transcription quantitative PCR (RT-qPCR) analyses. As expected, *busAA-AB* mRNA levels were lower in *gdpP* compared to WT-1 (Fig. 3D), consistent with previous findings that BusR represses *busAA-AB* expression under high c-di-AMP conditions (6). In the *gdpP*/*khpB-1* mutant, the levels of *busAA* and *busAB* RNA were significantly increased (9.3-fold and 7.5-fold, respectively), compared to the parent strain *gdpP*, despite both maintaining similarly high levels of c-di-AMP (Fig. 1C and 3D). The mRNA level of the transcriptional repressor *busR* remained unchanged.

To determine if the increased expression of BusAA-AB in *gdpP*/*khpB-1* led to increased uptake of glycine betaine, the intracellular level of this osmoprotectant was quantified using ultra-performance liquid chromatography–tandem mass spectrometry (UPLC-MS/MS). The *gdpP*/*khpB-1* mutant showed a 6.6-fold increase in intracellular glycine betaine compared to *gdpP* (Fig. 3E). Besides, as reported previously (6), the glycine betaine levels were much lower in *gdpP* than in WT-1 (Fig. 3E). Intracellular glycine betaine levels were also significantly higher in constructed *khpB* mutants of the laboratory *L. cremoris* strain MG1363 wild-type and its *gdpP* mutant, relative to their parent strains (Fig. 3F). Together, these findings suggest that loss of the RNA-binding protein KhpB leads to elevated intracellular glycine betaine levels by increasing *busAA-AB* RNA transcript and protein abundance.

### KhpB mutants have reduced cell size and increased secretion of Usp45 and OAS79_RS11150

**Fig 4:**
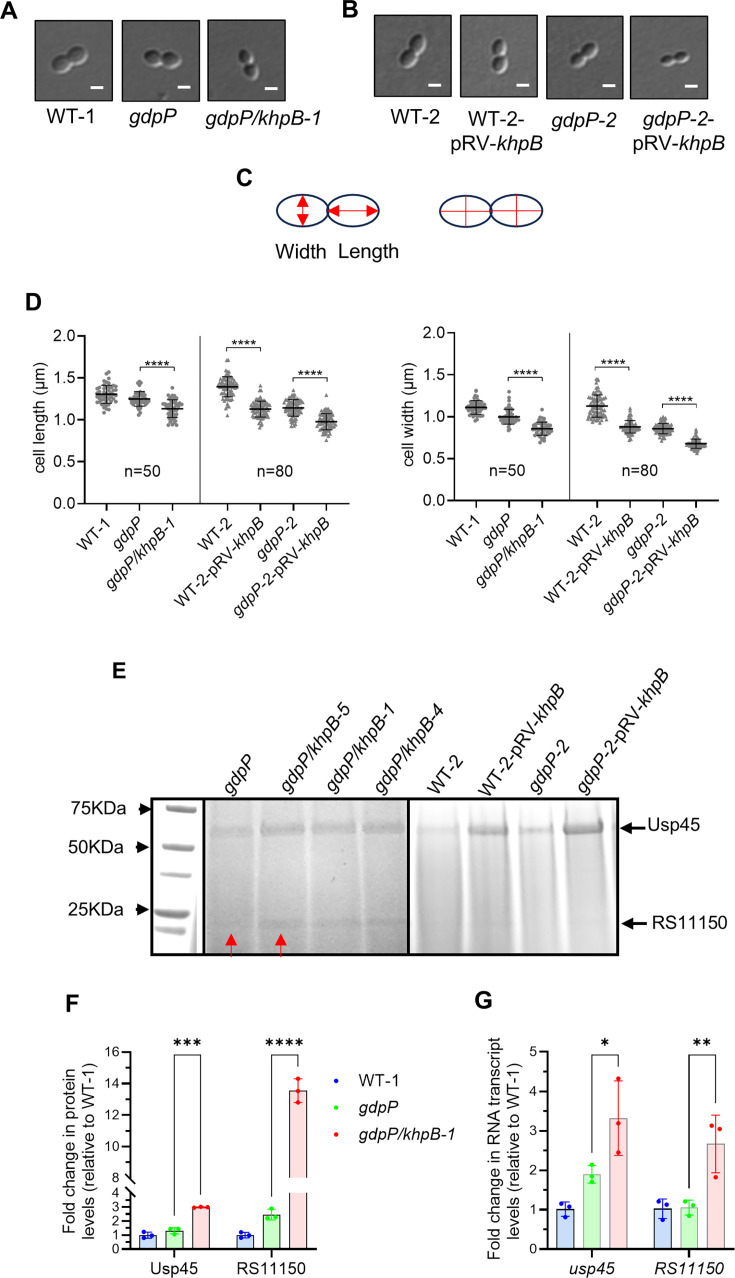
Involvement of KhpB in cell shape and size of *L. cremoris* strains. (**A and B**) Representative confocal microscopy images of WT-1 and its derivatives (**A**) and WT-2 and its derivatives (**B**). (**C**) Cells in the final stages of cell division were selected for the determination of length and width measurements. The left picture illustrates the measurement procedure, indicated by red arrows, while the right picture depicts the precise data collection process in a selected cellular state (phase 4), represented by red lines. (**D**) Cell length and width measurements of *L. cremoris* strains in the late dividing stage of the division cycle as shown in panels A and B. For WT-1 and derivative strains, 50 cells were measured. For WT-2 and derivative strains, 80 cells were measured. Data are shown as mean ± SD. One-way ANOVA followed by a multiple comparison test was used to evaluate significance (****P < 0.0001). (**E**) Usp45 (~60 kDa) and RS11150 (~20 kDa) levels in the spent supernatant of *L. cremoris* strains. Bands selected for protein identification are indicated by red arrows. (**F**) Abundance of proteins in WT-1, *gdpP*, and *gdpP/khpB-1* from the proteomics data set. Data are shown as mean ± SD. Statistical analysis was performed using two-way ANOVA followed by multiple comparison tests (**P < 0.001, ***P < 0.0001). (**G**) RT-qPCR analysis of RNA transcript levels of genes in WT-1, *gdpP*, and *gdpP/khpB-1*. Data are shown as mean ± SD. Two-way ANOVA followed by a multiple comparison test was used to evaluate significance (*P < 0.05, **P < 0.01).

Consistent with previous findings from studies on *S. pneumoniae* and *L. plantarum*, where *khpB* inactivation resulted in shorter cell lengths (39, 40) or smaller overall cell sizes (37, 41), we observed a similar phenotype in *L. cremoris*. Confocal microscopy showed that the cells of the *khpB* suppressor mutant (*gdpP*/*khpB-1*) were smaller than those of its parent strain *gdpP* (Fig. 4A). Similarly, targeted *khpB* inactivation in both the WT-2 and *gdpP-2* backgrounds resulted in visibly smaller cells (Fig. 4B). Quantitative measurements of at least 50 different cells confirmed these observations, with *khpB* mutants exhibiting significantly reduced cell length and width compared to their respective parent strains (*P* < 0.0001) (Fig. 4C and D).

Beyond morphological changes, *khpB* inactivation has also been found to influence protein secretion. Previous research showed that transposon insertions in *khpB* (also known as *ybdD*) resulted in an increased release of secreted proteins in *L. lactis*, including a heterologous secreted staphylococcal nuclease reporter and the dominant native *L. cremoris* secreted protein Usp45 (42, 43). To assess whether *khpB* inactivation similarly affected protein secretion in our *L. cremoris* strains, we analyzed the supernatant fractions from overnight cultures. We observed increased levels of Usp45 in both wild-type and *gdpP* mutant backgrounds following *khpB* inactivation (Fig. 4E). Another secreted protein, ~20 kDa in size, was observed to be more abundant in a *khpB* mutant than in *gdpP* in the industrial strain background (Fig. 4E). This protein band was excised and identified as OAS79_RS11150 (abbreviated as RS11150), which has an ortholog in *L. cremoris* MG1363 (LLMG_RS03925). RS11150 encodes a predicted lytic murein transglycosylase with a 26-amino acid secretion signal and a mature protein size of 20 kDa. It has a CW-7 domain (Pfam08230) for peptidoglycan binding and an RPF domain (cd13925) exhibiting lysozyme activity. Proteomics confirmed the increased level of RS11150 in *gdpP*/*khpB-1* (Fig. 3A and 4F), and RNA transcript levels were also found to be increased (Fig. 4G). However, unlike Usp45, the RS11150 band was less distinct on SDS-PAGE gels for laboratory strain WT-2 backgrounds.

These findings suggest that *khpB* inactivation not only alters cell morphology but also enhances the secretion of extracellular proteins. This could have significant implications for cell envelope remodeling and stress adaptation in *L. cremoris*.

### Mutation of *khpB* enhances milk acidification and growth of the high c-di-AMP *gdpP* mutant strain under salt stress

**Fig 5:**
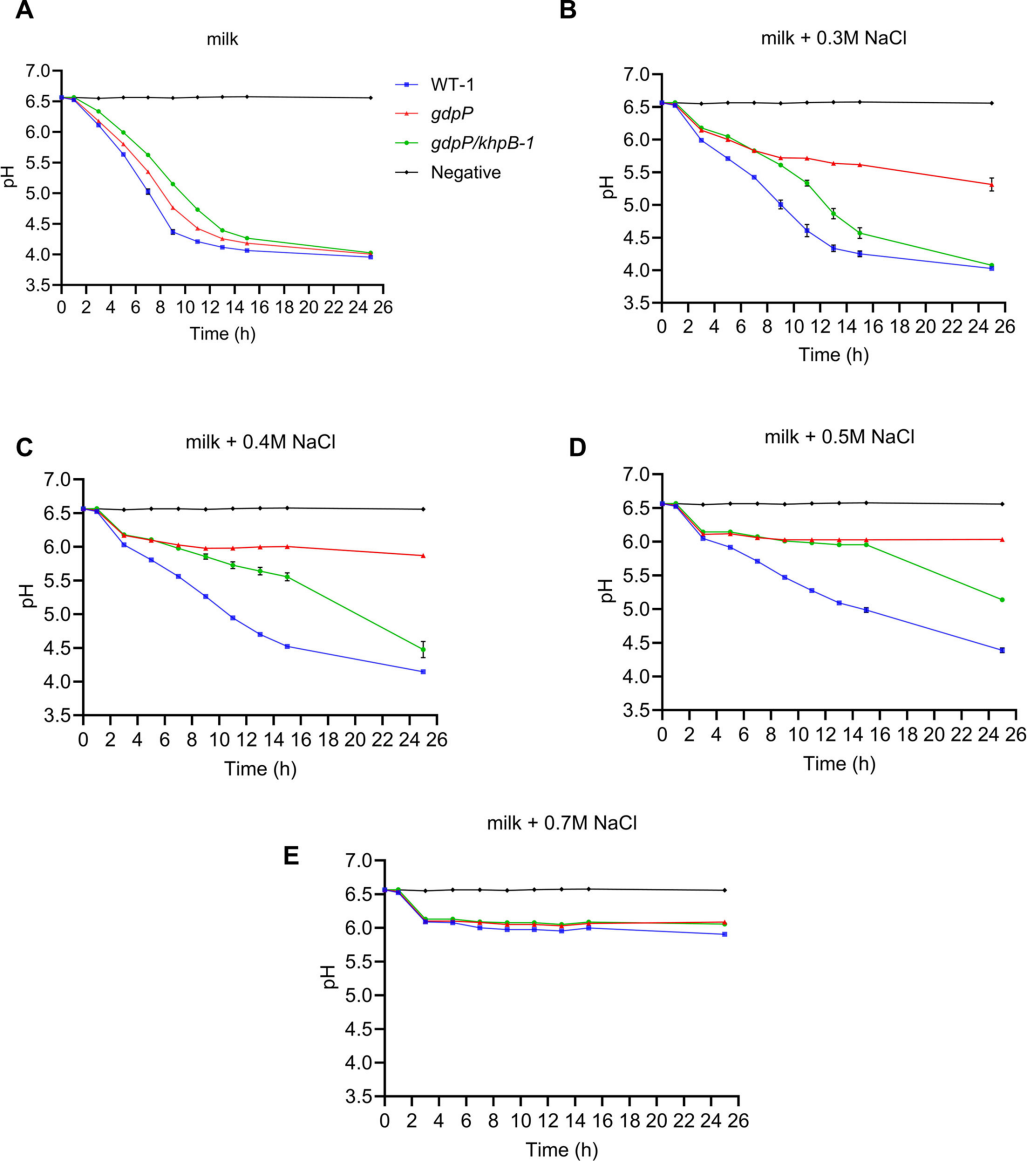
Acidification of milk with or without added NaCl by *L. cremoris*. The acidification ability of WT-1, *gdpP*, and *gdpP/khpB-1* in milk supplemented with (**A**) 0 M, (**B**) 0.3 M, (**C**) 0.4 M, (**D**) 0.5 M, and (**E**) 0.7 M NaCl was evaluated over 25 h by pH measurement. Uninoculated milk is marked as negative in the graphs. Data are shown as mean ± SD with three independent biological replications.

*L. cremoris* WT-1, an industrial cheese-making strain, contains the necessary genes for lactose and milk protein catabolism and is capable of growing in milk. During cheese-making, NaCl is added to slow down the acidification by the starter culture and to assist in whey removal and curd formation. We evaluated if the inactivation of *khpB* affects milk acidification with or without additional NaCl. The pH value of milk was monitored following inoculation of WT-1, *gdpP*, and *gdpP*/*khpB-1* strains. The *gdpP* strain was slower at acidifying milk compared to the wild type, while *gdpP*/*khpB-1* was even slower than its parent strain, *gdpP* (Fig. 5). Upon addition of 0.3, 0.4, and 0.5 M NaCl, the *gdpP* strain experienced the most significant reduction in acid production compared to WT-1 and *gdpP*/*khpB-1*. Notably, the *gdpP*/*khpB-1* mutant was more effective at acidifying milk than its parent *gdpP* at elevated NaCl levels (Fig. 5). However, the *gdpP*/*khpB-1* mutant was less effective at acidifying milk in the presence of NaCl compared to WT-1, suggesting that the suppressor mutant does not fully recover a NaCl-resistant phenotype, possibly due to retaining a high c-di-AMP level similar to its parent *gdpP* (Fig. 1C). We could not evaluate the effect of *khpB* inactivation in a low c-di-AMP background strain because WT-2 (*L. cremoris* MG1363) cannot grow in milk.

**Fig 6:**
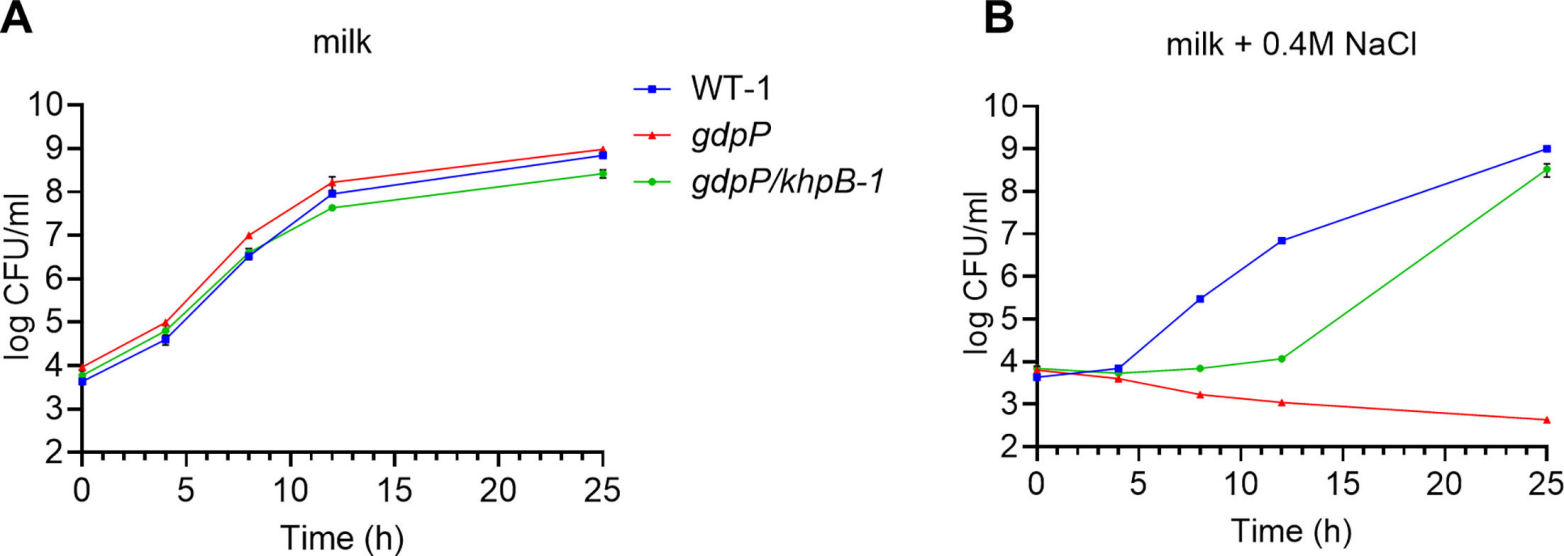
Growth of *L. cremoris* in milk with or without NaCl. CFU counts of WT-1, *gdpP*, and *gdpP/khpB-1* were determined in milk containing (**A**) 0 M or (**B**) 0.4 M NaCl at 0, 4, 8, 12, and 25 h by plating serial dilutions on LM17 agar. Data represent the mean ± SD from three independent biological replicates.

To determine if the acidification trend is associated with cell growth, strain colony-forming units (CFUs) were monitored over time in milk with or without 0.4 M NaCl. In milk without added NaCl, WT-1, *gdpP*, and *gdpP*/*khpB-1* strains displayed similar growth (Fig. 6A). With the addition of 0.4 M NaCl, *gdpP* showed a decline in viability over time, whereas *gdpP*/*khpB-1* was able to grow after an extended lag phase and reached similar cell numbers to WT-1 by 25 h (Fig. 6B). These findings indicate that *khpB* inactivation partially rescues acidification ability and growth in milk during salt stress in the *gdpP* mutant background.

## DISCUSSION

Osmotic regulation plays an important role in the robustness of industrial starter cultures, particularly in *Lactococcus* spp., when exposed to stress conditions. This study revealed that the inactivation of *khpB* significantly enhances osmoresistance of two different *L. cremoris* strains. Intriguingly, this enhancement in osmoresistance occurs without a reduction in c-di-AMP levels, suggesting a mechanism distinct from previously reported pathways controlled by this nucleotide signal (6, 15).

Previous research identified over 150 mutants in the c-di-AMP synthesis protein (CdaA) from laboratory *L. cremoris gdpP* mutants (15). Similarly, *cdaA* mutations (*n* = 27) were predominant in our screening of the industrial strain *gdpP*. In contrast to previous findings, we did not obtain mutations in *kupB* and *busR*, which encode c-di-AMP receptor proteins (6). Instead, a higher frequency of mutations was identified in genes related to cell wall function, including *khpB* (*n* = 6), cell wall pellicle gene cluster (*n* = 8), *ftsEX* (*n* = 2), and *greA* (*n* = 1). *L. cremoris*, lacking the cell wall pellicle, tends to form long chains, indicating a defect in cell separation similar to that seen in mutants lacking the major autolysin AcmA (28). Likewise, FtsX is also part of a multiprotein complex that regulates autolysin activity (44). We had previously identified a suppressor mutation in *ftsX* in an earlier salt-resistant suppressor screen of an *L. cremoris gdpP* mutant (6). Together, these results suggest a link between cell wall structure/homeostasis and osmoresistance in *L. cremoris*.

Glycine betaine is a key compatible solute that is imported by bacteria to respond to hyperosmotic stress (45, 46). In *L. cremoris*, the concentration of glycine betaine is regulated by c-di-AMP through its binding to the ATPase BusAA (11) or the transcriptional repressor BusR (6). Our investigation into osmotic regulation mechanisms in *khpB* mutants revealed that inactivation of *khpB* led to increased RNA transcript and protein levels of the glycine betaine transporter genes (*busAA-AB*), resulting in higher intracellular glycine betaine levels compared to the parent *gdpP* mutant strain. This suggests that KhpB may help regulate or compensate for the expression of *busAA-AB* under osmotic stress. Given that KhpB has been shown to bind RNA (29, 36), it is possible that the mRNA of *busAA-AB* may be a substrate of KhpB. Our experimental design evaluated gene expression, protein abundance, and intracellular glycine betaine levels under normal conditions, as osmotic stress is likely present even in the absence of added salt, allowing cells to dynamically balance water intake and loss (47).

Since *khpB* suppressor mutants maintain high c-di-AMP levels, the inhibition of *busAA-AB* transcription by BusR remains intact, suggesting that KhpB likely regulates *busAA-AB* transcript levels at a post-transcriptional stage. Recent studies also suggest that KhpB may be part of an RNA degradosome complex, interacting with RNA degradation enzymes such as PNPase, RNase E, and RNase J in *Mycobacterium* (48). Additionally, KhpB is part of a conserved gene operon (*rnpA-yidC-khpB*), and interactions between KhpB and the membrane protein insertase YidC have been reported in *S. pneumoniae* (49). It has been suggested that KhpB may regulate the RNA transcript stability of YidC substrates as they undergo coupled translation/membrane insertion (48). These findings support the hypothesis that KhpB could affect the stability of *busAA-AB* mRNA, though further investigation is needed to confirm this.

The KhpB-KhpA complex interacts with various RNAs, including mRNA, tRNA, and small regulatory RNA (sRNA), as identified by copurification assays. This complex modulates different RNAs across multiple pathways (28, 29). In *Clostridium difficile*, deleting *khpB* significantly alters the levels of many mRNA and small RNA transcripts, suggesting that KhpB could have a widespread regulatory effect, both enhancing and suppressing mRNA expression (28). Additionally, KhpA and KhpB downregulate transcription levels of the cell division protein FtsA (37) and affect the production of toxin A (36). These results highlight the role of KhpB and KhpA in controlling levels of specific RNAs, further supporting KhpB’s involvement in RNA-mediated regulation of cellular processes.

KhpA, which, like KhpB, also contains a potential RNA-binding KH domain, was identified as an interaction partner of KhpB through copurification and bacterial two-hybrid assays (28, 37, 49). The presence of both KhpA and KhpB is highly conserved in many bacteria, including *L. cremoris*, *L. plantarum*, *B. subtilis*, and *S. pneumoniae* (28). In *S. pneumoniae*, KhpA and KhpB have been shown to form both homo- and heterodimers and associate with sRNAs, suggesting a role in post-transcriptional regulation in the absence of canonical RNA chaperones such as Hfq or ProQ (28, 38). In *S. pneumoniae*, deletion of either *khpA*, *khpB*, or both genes leads to reduced cell size and growth defects, highlighting their coordinated role in cell elongation (37, 39). Similarly, in *L. cremoris*, we observed that inactivation of *khpB* also results in reduced cell dimensions, supporting a conserved function for KhpB in cell morphology. Whether KhpA plays a similar role in cell size and osmoresistance in *L. cremoris* remains to be investigated. In addition, KhpB binds the intracellular N-terminal region of MpgA via its Jag domain in *S. pneumoniae*, a finding we also confirmed in *L. cremoris* (Fig. S1). MpgA has recently been characterized as a muramidase that cleaves lipid-linked nascent peptidoglycan, facilitating its incorporation into the mature cell wall (43). Collectively, these observations suggest that KhpB may serve as a regulatory hub integrating RNA processing and cell wall metabolism.

Here we also confirm and extend the finding that KhpB is a regulator of secreted protein levels in *L. cremoris*. Loss of *khpB* resulted in significantly increased secretion of Usp45, which is the most abundant extracellular protein in *L. cremoris* (42), and RS11150, a peptidoglycan hydrolase with a lysozyme-like domain. These higher protein levels correlated with increased mRNA transcript levels, suggesting that KhpB controls the stability and/or translation of specific mRNAs encoding secreted proteins, consistent with previous observations in *L. lactis* where disruption of *ybdD* (*khpB* ortholog) led to increased Usp45 RNA transcript and protein levels (42). Interestingly, Usp45 shares 42% identity with PcsB from *S. pneumoniae*, a peptidoglycan hydrolase involved in cell division, and both proteins contain a cysteine, histidine-dependent amidohydrolase/peptidase domain (50). This further supports the idea that KhpB affects cell wall integrity and growth by modulating the secretion of key enzymes that maintain cell wall homeostasis.

In dairy fermentations, *Lactococcus* strains as starter cultures significantly contribute to the acidification and flavor of cheeses (51). However, the limited diversity of starter cultures used in cheese-making may increase strain susceptibility to environmental stressors such as phages, salt, and temperature fluctuations (52, 53). In this study, we observed that *khpB* inactivation in a wild-type (MG1363) background permitted growth under very high NaCl concentrations (up to 0.8 M NaCl). These findings highlight the potential of KhpB inactivation in enhancing osmoresistance in industrial strains. Understanding the genetic basis of osmoresistance is essential for optimizing starter cultures for stress resistance, including survival during freeze- or spray-drying and storage. This study provides new insights into genes involved in osmoresistance, offering potential new avenues for improving *Lactococcus* strains in dairy fermentations.

## MATERIALS AND METHODS

### Bacterial strains, growth conditions, and suppressor screening

The bacterial strains used in this study are shown in Table S1. *L. cremoris* strains were routinely grown at 30°C in M17 broth or agar (Difco, USA) with 0.5% (wt/vol) lactose (LM17) or glucose (GM17). When needed, 1.5% agar (Oxoid, UK) and/or 3 µg/mL erythromycin (Em) was added to the media. For salt suppressor screening, various agar media were used, including 1.33% (wt/vol) yeast extract (Oxoid) with 1% (vol/vol) 0.1 M K_2_HPO_4_ and 1.33% (wt/vol) lactose (LY) or glucose (GY); 10% (wt/vol) skim milk agar (Oxoid); 10% skim milk agar supplemented with 1% (vol/vol) M17 (SM); 4% (vol/vol) yeast extract (SY); or 5% (vol/vol) tryptone (ST). Plates were incubated for 1–3 days. Salt-tolerant suppressors of *gdpP* were obtained and verified as described before (15) using 0.25, 0.275, 0.3, 0.325, 0.35, and 0.45 M NaCl (ChemSupply, Australia) concentrations in the various media described above (LM17, LY, GY, SM, SY, ST, and skim milk agar). Subsequently, the pure colonies were confirmed by the serial dilution spotting method on the aforementioned agar plates as described previously (14). For *Escherichia coli* strains, derivatives of NEB5α and DHP_1 were grown at 37°C or 30°C, respectively, in Luria-Bertani (LB) broth (at 200–250 rpm) or on LB agar for 1–2 days. When needed, 50 and 100 µg/mL ampicillin were added to the media for *E. coli*.

### Suppressor mutant analysis and whole-genome sequencing

Genomic DNA was extracted using GenElute Bacterial Genomic DNA kit (Sigma-Aldrich, USA) for PCR or genetic manipulation. The genomic DNA for WGS was extracted as previously described (14). PCR products of the *cdaA* and *khpB* genes from salt suppressor mutants were analyzed by Sanger sequencing (Macrogen, South Korea). Whole-genome sequencing of WT-1, *gdpP*, and suppressors was conducted on the Illumina NovaSeq 6000 platform using 150 bp paired-end reads from a Nextera DNA XT library (Macrogen). For WT-1, a high-quality draft genome was generated using Oxford nanopore technology (ONT). Extracted DNA samples were prepared for sequencing following the protocol in the genomic sequencing kit SQK-LSK109 (ONT) with some modifications. Briefly, approximately 4 µg DNA was end-repaired and deoxyadenosine-tailed using the Ultra II end-repair module (New England BioLabs, USA). Sequencing adapters (ONT) were ligated using bound/T4 ligase (NEB). Libraries from the end-repair reaction and ligation steps were cleaned with AMPureXP beads (Beckman Coulter, USA). All incubation times for the end repair and ligation steps were extended. After cleaning, DNA was resuspended in 12 µL of elution buffer (ONT) before being combined with 37.5 µL of sequencing buffer (ONT) and 25.5 µL of loading beads and loaded on a MinION SpotON flow cell (FLO-MIN106). Sequencing was performed for 12 h with a MinION sequencer. Raw signals from ONT were used to recall bases using Guppy (version 4.2.2) (ONT). Reads from ONT were used to combine with paired-end sequencing reads from Illumina to create *de novo* assembly using Unicycler tool panel on Galaxy Australia (https://usegalaxy.org.au/). The final *de novo* assembly was submitted to Rapid Annotation using Subsystem Technology (https://rast.nmpdr.org/) for annotations. The SNPs of suppressor mutants were identified as previously described (6, 15) using Geneious Prime (version 2022.1.1; Biomatters Ltd., New Zealand).

### Extraction and quantification of the c-di-AMP level

The intracellular c-di-AMP level was extracted from *L. cremoris* using a previously established method (6) and quantified via liquid chromatography-coupled tandem mass spectrometry (LC-MS/MS; Shimadzu 8060, Shimadzu Corporation, Japan) using a Shim-pack Velox SP-C18 column (HSS PFP 1.8 µm, 2.1 × 150 mm; Shimadzu Corporation) as previously described (17). Mobile phase A was composed of 0.05% (vol/vol) formic acid (FA) in Milli-Q water, while mobile phase B was 0.05% (vol/vol) formic acid in 100% acetonitrile (ACN) (LC-MS grade). Ten microliters of the sample was injected at a flow rate of 0.3 mL/min at a column temperature of 40°C. Mobile phase A (95%) was used from 0 to 1 min, followed by a linear gradient from 95% to 50% for 10 min. The column was then washed with 90% mobile phase B for 3 min and then re-equilibrated with 95% mobile phase A for 2 min before the next injection. Azidothymidine (5 µM, Sigma-Aldrich) was used as an internal standard. C-di-AMP was detected using a triple-quadruple mass spectrometer equipped with an electrospray ionization source in negative ionization mode, monitoring multiple reaction transitions of *m*/*z* 657→134, 328, and 79 with mass transition at 134 used for quantification. Data were curated using LabSolutions Insight (version 3.2 SP1) and LabSolutions Postrun/QuantBrowser (version 5.95; Shimadzu Corporation). Biological triplicates were analyzed for each sample.

### Genetic manipulation

The bacterial strains, plasmids, and primers used in this study are shown in Table S1. To generate *khpB*-inactivated mutants in WT-2, a 610 bp internal fragment (from 14 to 623 bp) of the *khpB* gene from WT-2 was cloned into the PstI and XhoI sites in plasmid pRV300. The constructed plasmid pRV-*khpB* was transformed into *E. coli* NEB5α. pRV-*khpB* was electroporated into WT-2 and *gdpP-2* as previously described (14), and the transformants were plated out on an LM17 agar plate with 3 µg/mL Em and incubated at 30°C for 1–2 days. To excise the pRV-*khpB* plasmid, continuous subculturing was performed in GM17 broth for 3–5 days. Erythromycin-sensitive colonies, indicative of plasmid excision, were identified using replica plating on agar with or without Em.

### RNA preparation, cDNA synthesis, and RT-qPCR

Overnight cultures were diluted 1:100 in LM17 broth and grown to mid-log phase (OD_600_ ~0.6) at 30°C. RNA was extracted using the RNeasy Protect Bacteria Mini Kit (QIAGEN, Germany) with some modifications as previously described (6). For RNA stabilization, 500 µL of mid-log culture was mixed with 1,000 µL of RNA protect reagent in 2 mL tubes, vortexed for 5 s, and then incubated for 5 min at room temperature. The stabilized cells were harvested by centrifugation for 10 min at 5,000 × *g*. The cell pellet was resuspended in RNase-free Tris-EDTA buffer (Sigma-Aldrich) containing 15 mg/mL lysozyme (Merck, USA) and 10% Proteinase K (QIAGEN). DNA contamination was removed using an RNase-free DNase Set (QIAGEN). cDNA synthesis was performed using the SuperScript III First-Strand Synthesis SuperMix Kit (Invitrogen, USA). RT-qPCR was carried out using PowerUp SYBR Green Master Mix (Thermo Fisher Scientific, USA) on the Rotor-Gene Q PCR machine (QIAGEN).

*Lactococcus tufA*, a housekeeping gene encoding elongation factor TU essential for mRNA translation (6, 52, 54), was used as a control in this experiment. The expression of the reference gene *tufA* and test genes *busAB*, *busAA*, *busR*, *usp45*, and *RS11150* was assessed using the primers listed in Table S1. The amount of cDNA was normalized by using 0.5 µg of total RNA as input for reverse transcription. cDNA templates were diluted either 1:200 or 1:100 prior to their addition to the PCR mix. Each quantitative PCR run included a positive control (genomic DNA of WT-1 as template) and negative control (nuclease-free water as template substitute). Expression data were analyzed using the comparative CT method (2^−ΔΔCT^) based on results from biological triplicates (6), derived from three separately grown mid-log phase cultures. RNA extraction and cDNA synthesis were performed on these three independent samples. One-way analysis of variance (ANOVA) followed by a multiple comparison test was used for significant difference analysis using GraphPad Prism 9 (version 9.5.1).

### Proteomic analysis

*L. cremoris* WT-1, *gdpP*, and *gdpP*/*khpB-1* strains were grown overnight in LM17 broth and subcultured (1:100) into fresh LM17 medium. Cultures were incubated at 30°C until mid-log phase (OD_600_ ~0.7). Cells (2 mL) were collected by centrifugation (13,000 × *g*, 3 min, room temperature) and stored at −80°C until further processing. The samples were digested using the S-Trap Micro Spin Column Digestion Protocol and sent to Queensland Metabolomics and Proteomics for LC-MS/MS label-free proteomic analysis. Briefly, for normalization, 50 µg of total protein was lysed in 10% SDS lysis buffer with 20 mM dithiothreitol and incubated at 70°C for 1 h. After cooling, cysteine residues were alkylated with 40 mM iodoacetamide in the dark for 30 min. The lysate was then mixed with 5 µL of 27.5% phosphoric acid and 732 µL of S-Trap binding buffer, centrifuged at 13,000 × *g* for 8 min, and loaded onto S-Trap columns (4,000 × *g*, 1 min). After washing the column three times with 150 µL S-Trap binding buffer, trypsin digestion was performed by adding 1 µL of trypsin to 25 µL of 50 mM ammonium bicarbonate (pH 8) and performing overnight incubation at 37°C. Peptides were eluted sequentially with 40 µL of 5%, 50%, and 75% ACN in 0.1% FA and collected by centrifugation at 4,000 × *g* for 1 min. The top 110 µL of eluate was transferred to a 96-well plate and dried under vacuum. Peptides were resuspended in 20 µL of buffer A (5% ACN, 0.1% FA) before LC-MS/MS analysis.

Samples (2 µL) were injected into a Thermo Fisher UHPLC system coupled to an Exploris 480 mass spectrometer equipped with a FAIMS Pro interface. Peptides were first trapped on a C18 trap column (22 mm × 300 µm, 5 µm; Thermo Fisher Scientific) at 10 µL/min for 3 min, then separated on a nanoEase C18 resolving column (100 mm × 150 µm, 1.8 µm, 100 Å; Waters) using the following gradient: 8% for 4 min, then was increased linearly to 24% at 47 min, to 40% at 53 min, and to 95% at 57 min. The gradient was held constant for 1 min before returning to start condition at 8% over 1 min. FAIMS compensation voltages were set at −45 and −65 V. The electrospray voltage was held at 2.2 kV (positive mode), and the ion transfer tube was maintained at 295°C. Full MS scans were acquired using the Orbitrap (*m*/*z* 340–1110, resolution 120,000). MS/MS ions were measured in multiple isolation windows (350-470, 465-645, 640-1100 m/z) with 1 *m*/*z* overlap and quadrupole isolation. This result of proteomic analysis, performed in biological triplicates, is available in the supplemental material.

Proteomic data were analyzed using Spectronaut software (Biognosys AG, version 19.7.250203.62635) with a *Q* value cutoff of 0.05. Protein identification utilized the *L. cremoris* ASCC892185 reference proteome (GCA_030060875.1). Statistical analyses were conducted using Microsoft Excel. Fold changes in protein abundance were calculated relative to WT-1. For volcano plot analysis, the fold change cutoff was set at −1.5 ≤ fold change ≤ 1.5 [−0.58 ≤ log_2_(fold change) ≤ 0.58], with statistical significance defined as −log_10_(*P* value) ≥ 1.3 (*P* < 0.05).

### Quantification of glycine betaine

The extraction of glycine betaine was performed as described previously with some modifications (6). WT-1, *gdpP*, and *gdpP*/*khpB-1* were grown in LM17, while WT-2, WT-2-pRV*-khpB*, *gdpP-2*, and *gdpP-2-*pRV*-khpB* were grown in GM17. Overnight cultures were diluted 1:100 into 30 mL broth and incubated at 30°C until an OD_600_ of ~0.9 was obtained. Normalization was based on equivalent optical density. Cells were collected by centrifugation at 5,000 × *g* for 10 min and washed twice with 2 mL of 1:10 dilution of 0.01 M KPM buffer (pH 6.5). The cell pellets were resuspended in 1.6 mL ice-cold extraction buffer (methanol:acetonitrile:water = 2:2:1). Cell suspensions were transferred into 2 mL tubes with 0.5 mL equivalent of 0.1 mm zirconia/silica beads (Daintree Scientific, Australia). The cells were disrupted using a Precellys 24 homogenizer (Bertin Technologies) for five 30 s cycles, each with an interval of 1 min cooling on ice. Cell debris was removed by centrifugation at 17,000 × *g* for 15 min at 4°C, and the 1.2 mL supernatant was taken and dried by an RVC 2-18 CDplus Rotary Vacuum Concentrator. The dried pellets were resuspended in 500 µL MilliQ water. The level of glycine betaine was quantified using UPLC-MS/MS with a Shim-pack Velox SP-C18 column (HSS PFP 1.8 µm, 2.1 × 150 mm, Shimadzu Corporation), as described in the c-di-AMP quantification method above. Glycine betaine was detected with a triple-quadruple mass spectrometer equipped with an electrospray ionization source using multiple reaction monitoring transitions of *m*/*z* 118→59, 42, in negative ionization mode. The quantification was based on a standard curve derived from a pure betaine standard (Sigma-Aldrich). Biological triplicates were analyzed using this method. One-way ANOVA followed by a multiple comparison test was used for significant difference analysis using GraphPad Prism 9 (version 9.5.1).

### Cell size analysis with confocal microscope

For cell length and width analysis, cells from overnight cultures were subcultured (1 in 100) into GM17 or LM17 broth supplemented with 3 µg/mL Em when needed. Cells were incubated and collected in the early stationary phase (OD_600_ ~1.7). One microliter of cells was spotted on a thin 2% agarose gel patch. Images were acquired using a ZEISS LSM 900 Upright Laser Scanning Confocal Microscope with ×63 Plan-Apochromat DIC objective and two emerged channels (AF488 and T-PMT). The cell dimensions (length and width in micrometer) were quantified using the ZEN 2.6 Lite (blue edition) software. Approximately 50–80 cells from both WT-1 derivatives and WT-2 derivatives were measured, selecting only those in phase 4 of the division cycle, as detailed in previous studies (55, 56). One-way ANOVA followed by a multiple comparisons test was used for significant difference analysis using GraphPad Prism 9 (version 9.5.1)

### Supernatant protein precipitation and SDS-PAGE analysis

*L. cremoris* WT-2 derivatives were grown until an OD_600_ of ~1.2 was obtained in GM17 broth with or without Em, and WT-1 derivatives were grown in LM17 to reach the same OD and centrifuged at 10,000 × *g* for 5 min. Normalization was based on equivalent optical density. Proteins in the supernatant *L. cremoris* strains were precipitated using trichloroacetic acid (TCA), following a method similar to that described previously (52). The supernatant was filtered using a syringe filter (0.22 µm, Merck) and placed on ice for 5 min. Five percent (final concentration, vol/vol) ice-cold TCA (Sigma-Aldrich) was added to the filtered supernatant, allowing the proteins to precipitate on ice for 30 min. Then the precipitated proteins were centrifuged at 10,000 × *g* for 10 min at 4°C. The pellet was washed three times with 500 µL ice-cold acetone (Sigma-Aldrich) to remove residual TCA using the centrifugation conditions above. The washed pellet was then air-dried for 30 min. The dried pellet was resuspended in 30 µL of 5 mM NaOH and 10 µL of 4× SDS-PAGE loading buffer (200 mM Tris-HCl, pH 6.8, 8% SDS, 0.02% bromophenol blue, and 40% glycerol) containing 5% fresh β-mercaptoethanol (Sigma-Aldrich). The mixture was heated at 100°C for 5 min. The heated samples, along with the Precision Plus Protein All Blue Standards ladder (Bio-Rad, USA), were then loaded into a 4%–20% precast polyacrylamide gel (Bio-Rad) and separated at 200 V for 30–35 min. The gel was stained using Bio-Safe Coomassie G-250 Stain (Bio-Rad).

### Acidification and growth in milk

Milk acidification was performed as outlined previously (14). *L. cremoris* strains (WT-1 and derivatives) were grown overnight in LM17 at 30°C. Cells (5 mL) were collected (5,000 × *g*, 10 min), washed once with 1× phosphate-buffered saline (PBS, pH 7.4), and resuspended in 2.5 mL PBS. Resuspended culture (0.5 mL) was added to 43 mL full-cream UHT milk (Devondale; Murray Goulburn Co. Ltd, Australia). Inoculated milk tubes were incubated for 1 h at 30°C before adding 0, 0.3, 0.4, 0.5, and 0.7 M sterile NaCl (final volume was 50 mL, adjusted using sterile water). Samples were incubated at 30°C, and the pH was measured every 2–15 h and once more at 25 h. Uninoculated milk samples were included as a negative control. Biological triplicates were performed for each strain.

For evaluation of growth in milk, *L. cremoris* strains (WT-1 and derivatives) were grown overnight in LM17 at 30°C. Cultures were adjusted to an OD_600_ of 0.1 using PBS (pH 7.4). A 10  µL aliquot of the diluted culture was inoculated into 43 mL of full-cream UHT milk (Devondale, Murray Goulburn Co. Ltd) containing either 0 or 0.4 M sterile NaCl. The final volume was adjusted to 50 mL with sterile water. Samples were incubated at 30°C, and at 0, 4, 8, 12, and 25 h, 10  µL aliquots were serially diluted in 0.1% peptone water and plated onto LM17 agar. Plates were incubated at 30°C for 2–3 days before counting CFUs. All growth assays were tested in biological triplicate.

### assay

The bacterial adenylate cyclase two-hybrid (BACTH) system (Euromedex, France) uses the two split-adenylate cyclase complementary fragments T25 and T18 to evaluate the protein-protein interactions. When two target proteins interact, they bring together these fragments to reconstitute adenylate cyclase activity, leading to cyclic AMP production. This cyclic AMP then works with the catabolite activator protein to induce the expression of reporter gene *lacZ*. The resulting product, β-galactosidase, is both detectable and quantifiable. In our study, the nearly full-length MpgA encoding gene (4–1,636 bp) from WT-1 was genetically fused in frame to the T18 fragment in pUT18C, while the nearly full-length KhpB encoding gene (4–910 bp) or Jag_N domain only (4–247 bp) from WT-1 was fused to the T25 fragment in pKT25. The leucine zipper gene (*zip*) of GCN4, fused to the T25 and T18 fragments, was used as the positive control. Empty plasmids pUT18C and pKT25 with no inserts were used as the negative control. The plasmids carrying T18 and T25 fragments were cotransformed into *E. coli* DHP-1, and the β-galactosidase activity was tested on agar and broth as previously described (15). Biological triplicates were used for each sample.

## Data Availability

Proteomics raw data are available in the Mass Spectrometry Interactive Virtual Environment (MassIVE) database under accession no. MSV000098960 and ProteomeXchange under accession no. PXD067813). The draft genome is available in GenBank under BioSample SAMN31221605 and accession number NZ_CP109652.

## SUPPLEMENTAL MATERIAL

Data Set S1 aem.00914-25-s0001.xlsx Proteomics comparison between gdpP/khpB-1 and gdpP.

Data Set S2 aem.00914-25-s0002.xlsx Proteomics comparison between gdpP and WT-1.

Figure S1 aem.00914-25-s0003.TIF Bacterial two-hybrid analysis of KhpB interacting with MpgA.

Table S1 aem.00914-25-s0004.docx Strains, plasmids, and primers used in this study.

ASM does not own the copyrights to Supplemental Material that may be linked to, or accessed through, an article. The authors have granted ASM a non-exclusive, world-wide license to publish the Supplemental Material files. Please contact the corresponding author directly for reuse.
