# Supplementary figures and images for: Inactivation of KhpB (EloR/Jag) in Lactococcus cremoris increases uptake of the compatible solute glycine-betaine and enhances osmoresistance
Source: Appl Environ Microbiol. 2025 Sep 17;91(10):e00914-25. doi: 10.1128/aem.00914-25 (PMC12542702; doi:10.1128/aem.00914-25)

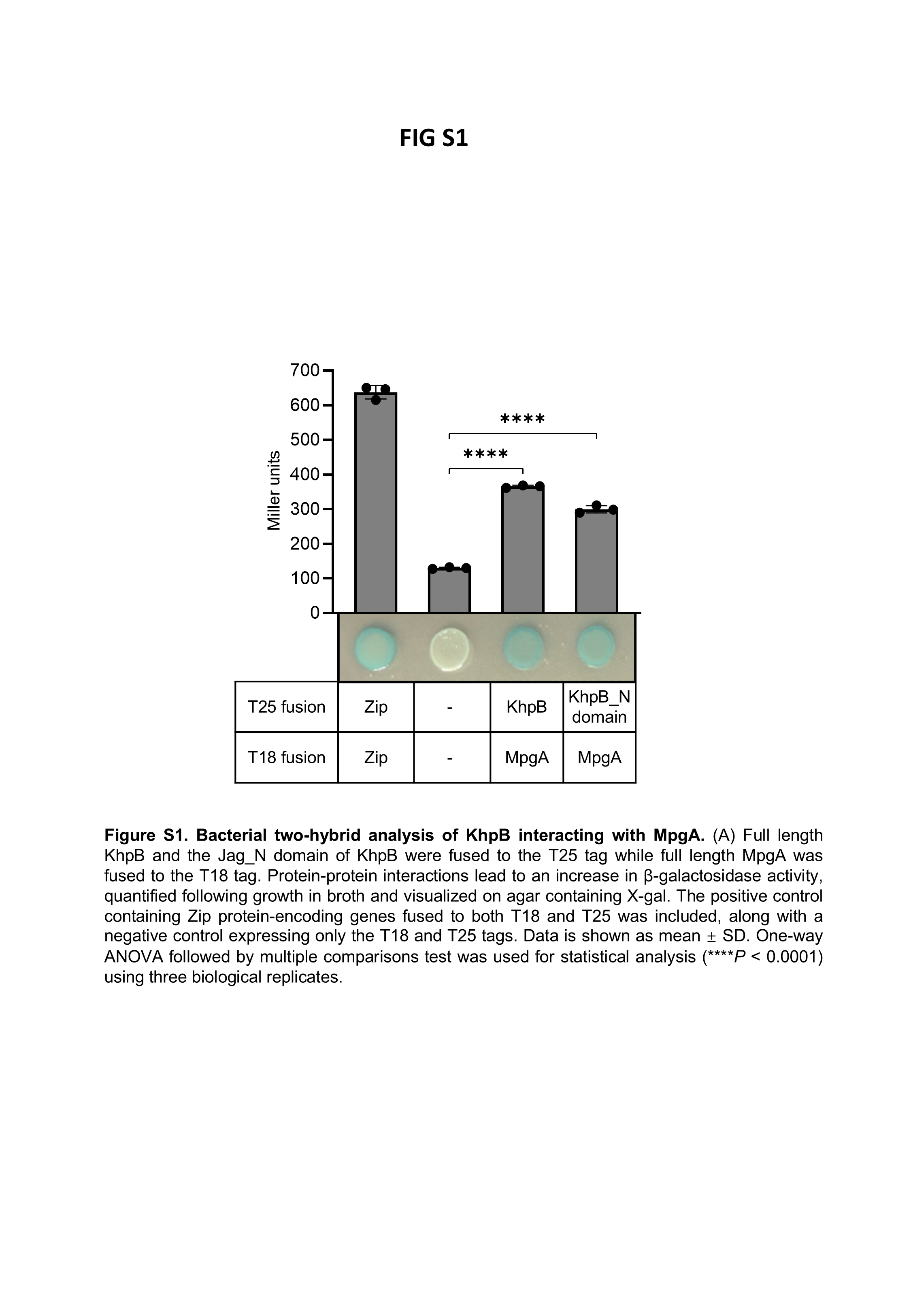

Supplement: Figure S1 — Bacterial two-hybrid analysis of KhpB interacting with MpgA. [file aem.00914-25-s0003.tif]
