# Supplementary material for: Inactivation of KhpB (EloR/Jag) in Lactococcus cremoris increases uptake of the compatible solute glycine-betaine and enhances osmoresistance
Source: Appl Environ Microbiol. 2025 Sep 17;91(10):e00914-25. doi: 10.1128/aem.00914-25 (PMC12542702; doi:10.1128/aem.00914-25)
Supplement: Table S1 — Strains, plasmids, and primers used in this study. [file aem.00914-25-s0004.docx]

Table S1: Strains, plasmids, and primers used in this study.

| ***L. lactis* strains** | **Properties** | **Phenotype** | **Source** |
| --- | --- | --- | --- |
| WT-1 | *L. lactis* subsp. *cremoris* industrial strain ASCC892185 |  | Dairy Innovation Australia Limited, Werribee, Victoria, Australia. Now property of Chr. Hansen. Denmark (Smith et al., 2012) |
| *gdpP* | Heat-resistant suppressor derived from ASCC892185 containing spontaneous mutations *gdpP*^E472Stop^^ and *potA*^D338N^ (encoding cyclic-di-AMP phosphodiesterase and spermidine/putrescine import ABC transporter ATP-binding protein, respectively, and genome sequenced in this study) | Heat resistant, salt sensitive, high c-di-AMP level | (Smith et al., 2012) (Zhu et al., 2016) |
| *gdpP/khpB-1,-2,-3^@^* | Salt-resistant suppressor derived from *gdpP* and contains the mutation *khpB*^K207fs*^ (genome sequenced in this study) | Salt-resistant, high c-di-AMP level | This study |
| *gdpP/khpB-4* | Salt-resistant suppressor derived from *gdpP* and contains the mutation *khpB*^E173fs*^ (genome sequenced in this study) | Salt resistant | This study |
| *gdpP/khpB-5^@^* | Salt-resistant suppressor derived from *gdpP* and contains the mutation *khpB*^G258fs*^ (sequenced the PCR product in this study) | Salt resistant | This study |
| *gdpP/khpB-6, -7* | Salt-resistant suppressor derived from *gdpP* and contains the mutation *khpB^D^*^198fs*^ and *rfbX*^W128stop^^ and another mutation *ytoL*^K243fs*^ on cytosolic protein containing multiple CBS domains (sequenced genome in this study) | Salt resistant | This study |
| *gdpP/khpB-8* | Salt-resistant suppressor derived from *gdpP* and contains the mutation *khpB*^R19stop^^ (sequenced genome in this study) | Salt resistant | This study |
| *gdpP/khpB-9, -10, -11, -12* | Salt-resistant suppressor derived from *gdpP* and contains the mutation *khpB*^K122fs*^ (sequenced genome in this study) | Salt resistant | This study |
| *gdpP/wpsF-1* | Salt-resistant suppressor derived from *gdpP* and contains the mutation *wpsF*^E149fs*^ (encoding galactofuranose transferase, genome sequenced in this study) | Salt resistant, high c-di-AMP level | This study |
| *gdpP/wpsF-2* | Salt-resistant suppressor derived from *gdpP* and contains the mutation *wpsF*^K93fs*^ (encoding galactofuranose transferase, genome sequenced in this study) | Salt resistant | This study |
| *gdpP/wpsF-3* | Salt-resistant suppressor derived from *gdpP* and contains the mutation *wpsF*^G174fs*^ (encoding galactofuranose transferase, genome sequenced in this study) and malolactic enzyme SfcA^Q342fs^ | Salt resistant | This study |
| *gdpP/wpsF-4* | Salt-resistant suppressor derived from *gdpP* and contains the mutation *wpsF*^S97stop ^^ (encoding galactofuranose transferase, genome sequenced in this study) | Salt resistant | This study |
| *gdpP/wpsD-1* | Salt-resistant suppressor derived from *gdpP* and contains the mutation *wpsD*^E27stop ^^ (encoding glycosyltransferase, genome sequenced in this study) | Salt resistant | This study |
| *gdpP/wpsE-1* | Salt-resistant suppressor derived from *gdpP* and contains the mutation *wpsE*^L223fs*^ (encoding glycosyltransferase, genome sequenced in this study) | Salt resistant | This study |
| *gdpP/wpsA-1* | Salt-resistant suppressor derived from *gdpP* and contains the mutation *wpsA*^A59V^ (encoding glycosyltransferase family GT2 protein, genome sequenced in this study) | Salt resistant | This study |
| *gdpP/ftsX-1* | Salt-resistant suppressor derived from *gdpP* and contains the mutation *ftsX*^A62D^ (encoding cell-division-associated ABC-transporter-like signalling protein and genome sequenced in this study) | Salt resistant, high c-di-AMP level | This study |
| *gdpP/ftsX-2* | Salt-resistant suppressor derived from *gdpP* and contains a 117bp deletion upstream of *ftsEX* | Salt resistant | This study |
| *gdpP/greA-1* | Salt-resistant suppressor derived from *gdpP* and contains a spontaneous mutation *greA*^E18Stop^ (encoding transcription elongation factor and genome sequenced in this study) | Salt resistant | This study |
| WT-2 | *L. lactis* subsp. *cremoris* MG1363, plasmid-cured |  |  |
| *gdpP-2* | Heat-resistant suppressor derived from MG1363 containing spontaneous mutations *gdpP*^K122Stop^ (encoding cyclic-di-AMP phosphodiesterase and sequenced previously) | Heat resistant, salt sensitive, high c-di-AMP level | (Smith et al., 2012) (Zhu et al., 2016) (Pham et al., 2021) |
| *gdpP-2*-pRV-*khpB* | *gdpP-2* containing integrated pRV300-*khpB* | Amp^r^, Em^r^ | This study |
| *gdpP-2*-pRV-*khpB* (excised) | pRV300-*jag* is excised from the chromosome of *gdpP*-2-pRV300-*khpB* | Amp^r^, Em^r^ | This study |
| MG1363-pRV-*khpB* | MG1363 containing integrated pRV300-*khpB* | Amp^r^, Em^r^ | This study |
| MG1363-pRV-*khpB* (excised) | pRV300-*jag* is excised from the chromosome of MG1363-pRV300-*khpB* | Amp^r^, Em^r^ | This study |
| ***E. coli* strains** | **Properties** | **Source** | |
| NEB5α | DH5α derivative, cloning host strain | New England Biolabs, USA | |
| DHP_1 | *cya* deficient derivative of DH1 used for the bacterial two-hybrid experiment | (Karimova et al., 2008) | |
| **Plasmids** | **Features** | **Antibiotic resistance** | **Source** |
| pRV300 | Non-replicative delivery vector pRV300 in *L. lactis*. Replicates in *E. coli*. | Amp^r^, Em^r^ | (Leloup et al., 1997) |
| pRV-*khpB* | pRV300 with 610bp internal fragment of *khpB* for gene inactivation | Amp^r^, Em^r^ | This study |
| pUT18C | Expresses the T18 fragment of *Bordetella pertussis* adenylate cyclase (CyaA) | Amp^r^ | Euromedex, France |
| pUT18C-*zip* | The leucine zipper of GCN4 is fused in frame to the T18 fragment (inserted between the KpnI and EcoRI sites) | Amp^r^ | Euromedex, France |
| pUT18C-*mltG* | *L. lactis* WT-1 *mltG* gene (4bp to 1636 bp) was fused in frame with T18 fragment. | Amp^r^ | This study |
| pKT25 | Expresses the T25 fragment of *Bordetella pertussis* adenylate cyclase (CyaA) | Kan^r^ | Euromedex, France |
| pKT25-*zip* | The leucine zipper of GCN4 is fused in frame to the T25 fragment (inserted within the KpnI site) | Kan^r^ | Euromedex, France |
| pKT25-*khpB* | *L. lactis* WT-1 *khpB* gene (4bp to 910 bp) is genetically fused in frame to the T25 fragment. | Kan^r^ | This study |
| pKT25-*jag domain* | *L. lactis* WT-1 *jag* domain (4 bp to 247 bp) is fused in fame with the T25 fragment | Kan^r^ | This study |
| **Primer name** | **Sequence (5' to 3')** | **Function** | **Source** |
| 0448-F(A) | GTGCTTGGCGAATGACTTG | Amplification and sequencing of *cdaA* from WT-1 derivatives | (Zhu et al., 2016) |
| 0448-R | CGGTATATACTTCACCAGATTGG | Amplification and sequencing of *cdaA* from WT-1 derivatives | (Zhu et al., 2016) |
| KhpB-F-PstI | TTGCTGCAGGAGAAGCTAAGGTACAGGTCG | Amplification and sequencing of *khpB* from WT-1 derivatives | This study |
| KhpB-R-XhoI | TTCCTCGAGGGTTGAAAGTAGCTCCACC | Amplification and sequencing of *khpB* from WT-1 derivatives | This study |
| KhpB-For-Pst-pKT25 | ATA**CTGCAG**CTATTTTTACTGGTGAAACGGTC | Used to introduce *khpB/jag-domain* into BACTH plasmid pKT25 | This study |
| KhpB-Rev-Kpn | ATT**GGTACC**AAAATCTCTTTAGAGACAACAATATA | Used to introduce *khpB* into BACTH plasmid pKT25 | This study |
| KhpB domain-Rev-Kpn | ATT**GGTACC**ACGGATTGTGATTTAGGAACG | Used to introduce *jag domain* into BACTH plasmid pKT25 | This study |
| MpgA-For-Pst | ATA**CTGCAG**TGACAAAGAAACAACTGAATTTTCTC | Used to introduce *mpgA* into BACTH plasmid pUT18C | This study |
| MpgA-Rev-Kpn | ATT**GGTACC**AATTTACTTAATTTATCATTCACATA | Used to introduce *mpgA* into BACTH plasmid pUT18C | This study |
| pUT18C-F | GAAGTTCTCGCCGGATGTACTG | Used to sequence inserts in pUT18C | This study |
| pUT18C-R | GCTTAACTATGCGGCATCAG | Used to sequence inserts in pUT18C | This study |
| pKT25-F | GCCGCATCTGTCCAACTTCC | Used to sequence inserts in pKT25 | This study |
| pKT25-R | GCTGCAAGGCGATTAAGTTG | Used to sequence inserts in pKT25 | This study |
| Vlac1 | GTTGAATAACACTTATTCCTATC | Flanking pTCV-*lac* cloning sites, used to sequence inserts in pTCV-*lac* | (Poyart et al., 1997) |
| Vlac2 | CTTCCACAGTAGTTCACCACC | Flanking pTCV-*lac* cloning sites, used to sequence inserts in pTCV-*lac* | (Poyart et al., 1997) |
| M13F | GTAAAACGACGGCCAG | Flanking pGh9 cloning sites, used to confirm integration | Universal primer |
| Bluescript SK | CGCTCTAGAACTAGTGGATC | Flanking pGh9 cloning sites, used to confirm integration | Universal primer |
| KhpB-KO-F | CTA**CTGCAG**CTGGTGAAACGGTCGAAG | Used to introduce a 610bp internal fragment of *khpB* from MG1363 into pRV300 | This study |
| KhpB-KO-R | CTT**CTCGAG**ATTTTCCCATGCTTCCC | Used to introduce a 610bp internal fragment of *khpB* from MG1363 into pRV300 | This study |
| FUP | GTAAAACGACGGCCAGTG | Flanking pRV300 cloning sites, used to confirm integration | This study |
| RUP | CAGGAAACAGCTATGAC | Flanking pRV300 cloning sites, used to confirm integration | (Pham et al., 2018) |
| KhpB-up | CCATACAAGATTATTGCAGC | Primer upstream of *khpB* from MG1363 derivatives, used to confirm integration | This study |
| TufA-F | GGTAGTTGTCGAAGAATGGAGTGTGA | Housekeeping gene for RT-qPCR | (Pham et al., 2018) |
| TufA-R | TAAACCAGGTTCAATCACTCCACACA | Housekeeping gene for RT-qPCR | (Pham et al., 2018) |
| BusAA-F | GGTTTGTCCGGTTCAGGGAA | Amplification of *busAA* by RT-qPCR | This study |
| BusAA-R | ACGGCGAACTTGAAGCAAAT | Amplification of *busAA* by RT-qPCR | This study |

| BusAB-F | TCATTTGGTTCGACTGCCCG | Amplification of *busAB* by RT-qPCR | This study |
| --- | --- | --- | --- |
| BusAB-R | GCAGCTAGTACTCCACGACC | Amplification of *busAB* by RT-qPCR | This study |
| BusR-F | CCCTGTTCATGGAAGTGGTGT | Amplification of *busR* by RT-qPCR | This study |
| BusR-R | TGCATGATTTGGGTTCGCAAG | Amplification of *busR* by RT-qPCR | This study |
| Usp45-F | GCTGCTCCTGGTGTTATCGT | Amplification of *usp45* by RT-qPCR | This study |
| Usp45-R | CGTTCATGTCCCCACCAAGT | Amplification of *usp45* by RT-qPCR | This study |
| RS11150-F | ACGAGCCAAACTTCTGGACA | Amplification of *llmg_0760* by RT-qPCR | This study |
| RS11150-R | ATTGGAAGGCACCGTAAGCA | Amplification of *llmg_0760* by RT-qPCR | This study |

* fs = frameshift; ^ Stop = stop codon; @= *gdpP/khpB-3* and *gdpP/khpB-5* were identified by PCR with Sanger sequencing and other SNPs were identified by WGS.

Amp^r^ means ampicillin resistance. Em^r^ means erythromycin resistance. Kan^r^ means kanamycin resistance.

**References**

SMITH, W. M., PHAM, T. H., LEI, L., DOU, J., SOOMRO, A. H., BEATSON, S. A., DYKES, G. A. & TURNER, M. S. 2012. Heat resistance and salt hypersensitivity in *Lactococcus lactis* due to spontaneous mutation of *llmg_1816* (*gdpP*) induced by high-temperature growth. Appl Environ Microbiol, 78, 7753-9.

ZHU, Y., PHAM, T. H., NHIEP, T. H., VU, N. M., MARCELLIN, E., CHAKRABORTTI, A., WANG, Y., WAANDERS, J., LO, R., HUSTON, W. M., BANSAL, N., NIELSEN, L. K., LIANG, Z. X. & TURNER, M. S. 2016. Cyclic-di-AMP synthesis by the diadenylate cyclase CdaA is modulated by the peptidoglycan biosynthesis enzyme GlmM in *Lactococcus lactis*. Mol Microbiol, 99, 1015-27.

PHAM, H. T., NHIEP, N. T. H., VU, T. N. M., HUYNH, T. N., ZHU, Y., HUYNH, A. L. D., CHAKRABORTTI, A., MARCELLIN, E., LO, R., HOWARD, C. B., BANSAL, N., WOODWARD, J. J., LIANG, Z. X. & TURNER, M. S. 2018. Enhanced uptake of potassium or glycine betaine or export of cyclic-di-AMP restores osmoresistance in a high cyclic-di-AMP *Lactococcus lactis* mutant. PLoS Genet, 14, e1007574.

POYART, C. & TRIEU-CUOT, P. 1997. A broad-host-range mobilizable shuttle vector for the construction of transcriptional fusions to β-galactosidase in Gram-positive bacteria. FEMS Microbiology Letters, 156, 193-198.
